# The effects of isolated ankle strengthening and functional balance training on strength, running mechanics, postural control and injury prevention in novice runners: design of a randomized controlled trial

**DOI:** 10.1186/1471-2474-15-407

**Published:** 2014-12-04

**Authors:** Jennifer Baltich, Carolyn A Emery, Darren Stefanyshyn, Benno M Nigg

**Affiliations:** Human Performance Laboratory, Faculty of Kinesiology, University of Calgary, Calgary, Alberta Canada; Sport Injury Prevention Research Centre, Faculty of Kinesiology, University of Calgary, Calgary, Alberta Canada

**Keywords:** Prevention, Running injury, Ankle strength, Neuromuscular training, Exercise intervention, Randomized controlled trial

## Abstract

**Background:**

Risk factors have been proposed for running injuries including (a) reduced muscular strength, (b) excessive joint movements and (c) excessive joint moments in the frontal and transverse planes. To date, many running injury prevention programs have focused on a “top down” approach to strengthen the hip musculature in the attempt to reduce movements and moments at the hip, knee, and/or ankle joints. However, running mechanics did not change when hip muscle strength increased. It could be speculated that emphasis should be placed on increasing the strength of the ankle joint for a “ground up” approach. Strengthening of the large and small muscles crossing the ankle joint is assumed to change the force distribution for these muscles and to increase the use of smaller muscles. This would be associated with a reduction of joint and insertion forces, which could have a beneficial effect on injury prevention. However, training of the ankle joint as an injury prevention strategy has not been studied. Ankle strengthening techniques include isolated strengthening or movement-related strengthening such as functional balance training. There is little knowledge about the efficacy of such training programs on strength alteration, gait or injury reduction.

**Methods/Design:**

Novice runners will be randomly assigned to one of three groups: an isolated ankle strengthening group (strength, n = 40), a functional balance training group (balance, n = 40) or an activity-matched control group (control, n = 40). Isokinetic strength will be measured using a Biodex System 3 dynamometer. Running kinematics and kinetics will be assessed using 3D motion analysis and a force platform. Postural control will be assessed by quantifying the magnitude and temporal structure of the center of pressure trace during single leg stance on a force platform. The change pre- and post-training in isokinetic strength, running mechanics, and postural control variables will be compared following the interventions. Injuries rates will be compared between groups over 6 months.

**Discussion:**

Avoiding injury will allow individuals to enjoy the benefits of participating in aerobic activities and reduce the healthcare costs associated with running injuries.

**Trial registration:**

Current Controlled TrialNCT01900262.

**Electronic supplementary material:**

The online version of this article (doi:10.1186/1471-2474-15-407) contains supplementary material, which is available to authorized users.

## Background

In the 1960s, research started to demonstrate a strong relationship between physical inactivity and a variety of diseases and health conditions, including coronary heart disease, hypertension, obesity and osteoporosis. Several studies found that participation in regular aerobic exercise does in fact reduce the risk of suffering from such conditions[1–6]. With the increasing awareness of the benefits of physical activity, a more health conscious general population expressed interest in exercise. Recreational running, with its accessibility and low monetary cost, has grown increasingly popular among the general population as a primary form of exercise[7]. However, the increase in popularity of running has also led to an increase in running-related injuries. Previous retrospective and prospective running injury studies have found the yearly injury incidence proportion to range from 26-85%[8–13]. The effects of running injuries include short-term and long term pain and discomfort. Short-term pain and discomfort would be due to the immediate effects of the injury. Long term effects may include a reduction of physical activity, osteoarthritis following acute injury, and increased health care costs[14, 15]. This highlights the impetus for further investigation into running injury prevention, as this is a growing concern for the general population not only from a running injury perspective, but also from a health standpoint.

A variety of intrinsic risk factors that are inherent to the runner have been identified for running injury. Some of the most commonly studied intrinsic variables include muscle strength and joint kinematics and kinetics during running[16–18].

### Muscle strength

It has been found that weak plantar flexor strength is associated with an increased incidence of Achilles tendinopathy, one of the most common overuse running injuries[16]. Furthermore, decreased muscular strength, particularly at the quadriceps and hamstrings, has been associated with increased incidence of patellofemoral pain syndrome, another common overuse running injury[17]. Reduced hip abduction strength has also been found in subjects suffering from patellofemoral pain syndrome and has generally been found to be a strong predictor of lower extremity injury risk[19, 20].

### Joint kinematics and kinetics

Research examining joint kinematic and kinetic variables during running has found that increased hip adduction and knee internal rotation are risk factors for the development of iliotibial band syndrome in runners[18]. Furthermore, it is thought that abnormal movement of the tibia and femur in the frontal and transverse planes affect patellar tracking and, therefore, increase the risk of suffering from patellofemoral pain syndrome[21]. This has been seen in runners with patellofemoral pain syndrome who demonstrated increased ankle eversion velocities and altered joint coupling mechanics[22]. It also has been found that runners with patellofemoral pain demonstrate increased knee joint angular impulse in the frontal plane compared to healthy runners[23].

Most of the recent running injury prevention studies have focused on hip strengthening protocols with the hope of reducing excessive joint movements and moments[24, 25]. The strengthening of the hip joint musculature assumes a “top down” approach where it is thought that improving strength at the hip will not only reduce movements and loading at the hip, but also at the more distal knee and ankle joints[26–28]. However, hip strengthening protocol interventions demonstrated that an increase in hip muscular strength was not accompanied by a reduction in excessive joint movements or moments in the frontal and transverse planes during running[24]. One potential explanation for these findings would be that the strengthening protocols were focusing on the proximal hip joint. It may be that a “ground up” approach should be used, in which specific ankle strengthening would be the primary focus of training. Such an approach would hypothesize that changes at the ankle joint will influence the mechanics of the more proximal knee and hip joints[27, 29]. Biomechanical injury prevention models focus on adjusting both the external and internal loads on the body[30]. In this sense, interventions aim to reduce the loads below the injury threshold or increase the body’s ability to tolerate the load[30]. It has been proposed that isolated ankle strengthening increases the strength of the small muscles surrounding the ankle joints, which are more adept for making quick postural adjustments without excessive force[31]. The use of these small stabilizing muscles rather than the larger muscles with longer lever arms may increase joint stability and reduce the loading at the joint and insertions of the larger muscles surrounding the ankle joint[31]. As a result, increased ankle musculature strength may not only increase the body’s ability to tolerate the load through an increased muscle ultimate strength but may also reduce the internal loads at the joint and at the muscle insertions below the injury threshold[31].

Another potential explanation for the lack of kinematic and kinetic changes found with hip musculature strengthening may be that the strengthening protocols used were not functional for an activity such as running. The principle of specificity dictates that training protocols should mimic the movements of the particular sport as closely as possible in order for the body to adapt appropriately[32]. Clinicians accept the importance of functional training and incorporate functional movements into training protocols, however there is a paucity of literature evaluating such functional training programs[33, 34]. Some hip strengthening protocols primarily focus on open chain, non-weight bearing exercises to strengthen the hip and thigh musculature[24]. Running is a closed chain, weight-bearing exercise and, therefore, training that does not incorporate similar mechanics that replicate the running movement may not alter running kinematics and kinetics. The only closed chain, weight-bearing exercise used by Willy and Davis was the single leg squat movement re-education protocol. The only movement differences found were those during the single-leg squat rather than during running. Similar results have been found previously indicating that isolated strengthening programs may not affect movement mechanics[35]. In order to alter lower extremity movement mechanics, training protocols may need to incorporate functional movements in order to improve neuromuscular control at the joint.

One form of training that may be able to accomplish this functional requirement is the incorporation of an unstable device into the training protocol. The appropriate training program using an unstable device should be able to incorporate functional closed chain, weight-bearing exercises that replicate the running movement to possibly increase strength and reduce excessive joint movements and moments during running. Functional balance training has traditionally been used in ankle injury rehabilitation programs with patients suffering from chronic ankle instability and repeated ankle sprains[36, 37]. One main goal of rehabilitation programs was to increase the strength of the ankle joint following injury. Research examining the effects of such training on lower extremity strength has shown successful increases in muscle strength[38, 39]. In recent years, functional balance training protocols have been utilized for the prevention of lower extremity injury in sport for healthy, physically active participants. Multifaceted training programs have seen positive results with respect to the reduction of excessive joint movements and moments as well as the incidence of acute and overuse lower extremity injuries in sport[40–42]. The same group that had utilized a multifaceted training program to reduce knee valgus in high school athletes went on to investigate the effects of the balance training portion of their protocol on knee valgus during landing[43]. For the same duration and intensity of training that was used in the multifaceted approach, it was found that balance training alone reduced hip adduction and ankle eversion angles during landing from a drop vertical jump. Similar results have been found in other functional balance training programs where frontal and transverse plane movements and joint moments have successfully been reduced. Even more importantly, functional balance training has successfully reduced the incidence of acute and overuse injuries in sports such as soccer, volleyball and basketball where non-contact ACL injuries and patellofemoral pain syndrome are common[42, 44–50]. However, such an intervention has yet to be evaluated in a recreational running setting.

#### Rationale

In summary, recreational running has grown in popularity as a form of aerobic exercise. Despite the health benefits associated with running, this steep increase in participation has inevitably led to an increase in the quantity of running injuries. Intrinsic risk factors have been associated with an increased risk of suffering from injuries, including reduced muscular strength and excessive lower extremity joint movements and joint moments in the transverse and frontal planes. Current injury prevention methods have focused on strengthening the hip and thigh musculature in the hopes of reducing excessive joint movements and moments. However, these strengthening interventions have not been successful for influencing running kinematics and kinetics and have yet to be tested in a prospective injury analysis. One potential reason for the lack of success with altering movement mechanics may be that the strengthening protocols are focusing primarily on the proximal joint. Another possible explanation is that the strengthening protocols were not functional for running, and thus, did not impact movement mechanics. Therefore, the purpose of this study is to compare the effects of isolated ankle joint strengthening and functional balance training with an activity-matched control group on lower extremity strength, running mechanics, postural control and the incidence of injury in healthy novice runners.

#### Objectives

To compare changes in (1) ankle, knee, and hip isokinetic strength, (2) lower extremity running kinetics and kinematics, and (3) postural control in healthy adult novice runners participating in an 8-week isolated ankle strengthening program or an 8-week functional balance training group to an activity-matched control group. Additionally, (4) injury rates in healthy adult novice runners participating in an 8-week isolated ankle strengthening program or an 8-week functional balance training group will be compared to those in an activity-matched control group.

#### Hypotheses

All hypotheses are made based on comparisons between each of the training groups and the control group.

1. Isolated ankle strengthening will increase ankle isokinetic strength but not strength at the knee or hip joints.
2. Isolated ankle strengthening will significantly decrease joint movements and joint moments in the frontal and transverse planes at the ankle during over ground and treadmill running.
3. Functional balance training will significantly increase ankle and knee and hip isokinetic strength.
4. Functional balance training will significantly decrease joint movements and joint moments in the frontal and sagittal planes at the ankle, knee and hip joints during over ground and treadmill running.
5. Functional balance training will significantly reduce the center of pressure movement magnitude, increase the regularity of the center of pressure time series and improve performance on the Star Excursion Balance Test.
6. Injury rates will be lower for the functional balance training group and the isolated ankle strengthening group compared to the activity matched control group.

## Methods/Design

### Study design

**Figure 1:**
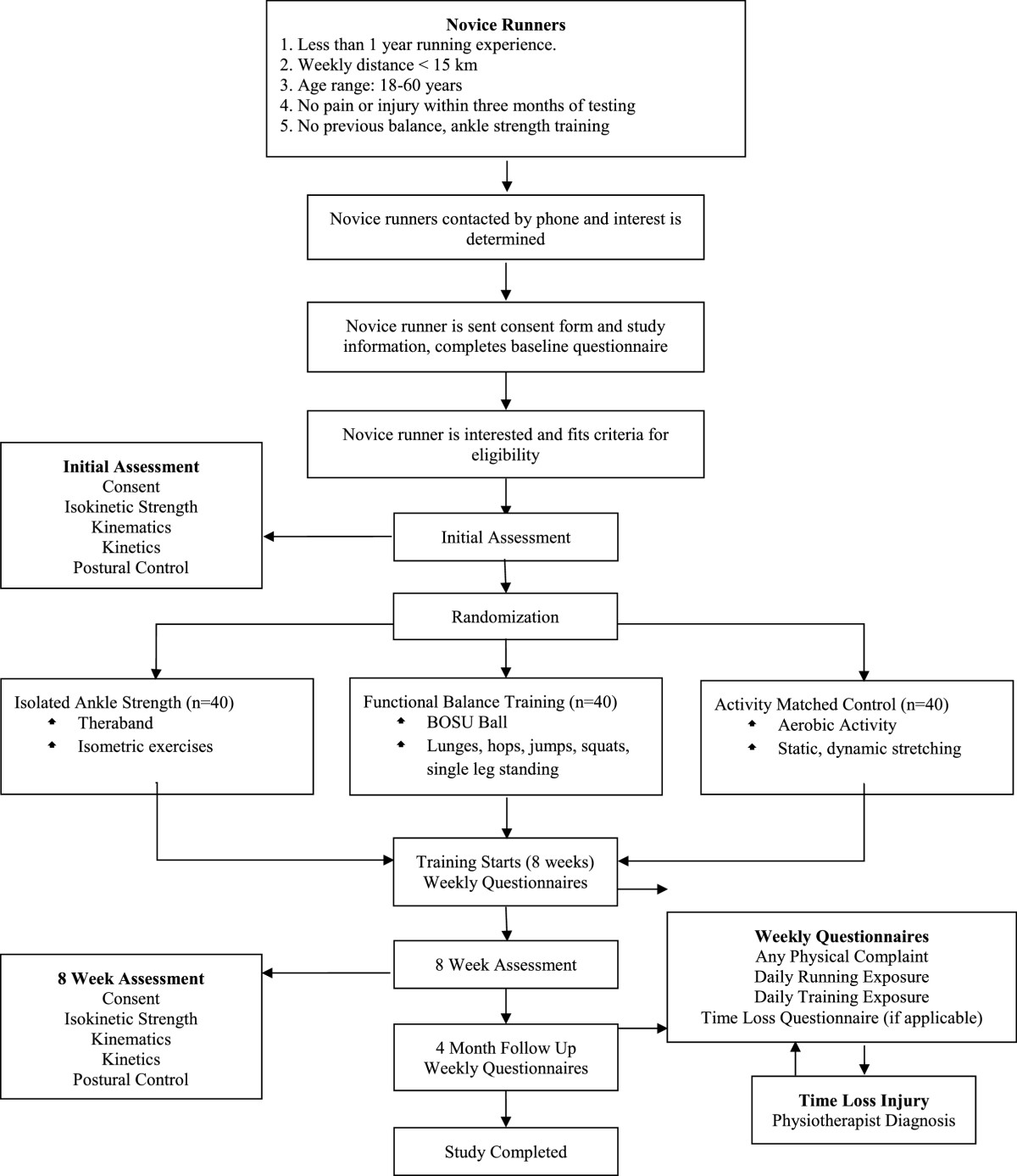
**Study design protocol.**

This is a randomized controlled trial (RCT). In total, 120 novice runners will be recruited. Subjects will be randomly assigned to one of three groups: an isolated ankle strengthening group (n = 40), a functional balance training group (n = 40) or an activity matched control group (n = 40). The study design and flow can be seen in Figure 1. Research ethics approval was obtained from the University of Calgary’s Conjoint Health Research Ethics Board (Ethics ID: REB13-0153).

### Target population

The target population for this research project is novice recreational runners. Novice runners have been chosen for this study as they may be the most malleable for training effects. They have also been shown to have a higher incidence or running-related injuries than experienced runners[51–54]. A novice recreational runner will be identified as an individual with less than one year of running experience that runs at least twice a week for a total weekly distance greater than 5 kilometers as their main physical activity[54]. An age range of 18 – 60 years of age will be used for subject inclusion criteria. Inclusion criteria include the lack of any neurological abnormalities or any lower extremity injury or pain within the three months prior to testing and subjects should not have extensive prior experience with isolated joint strength training functional balance training within the last year.

### Sample size

Exploratory power analyses were completed for multiple biomechanical variables that will be tested in this study. Sample size was determined based on a sample size calculation using previously published data on the effects of balance training on the 95% ellipse area of the center of pressure as this was the largest reasonable sample size needed for the variables in this study[37]. It was found that in order to achieve 80% statistical power with an α = 0.05, 32 subjects would be needed to detect a change between training group and the control group with respect to the change in the 95% ellipse area following training with an effect size of 0.71. Assuming a 20% drop out rate, 40 subjects will be recruited for each of the groups in this project. Due to the sample size, the injury portion of this study will be a pilot RCT for developing a running injury prevention program. The information found in this study regarding the incidence of injury in each group will be critical for developing future RCTs investigating the impact of these interventions on running injury prevention.

### Randomization procedure

In order to protect against selection bias, subjects will be randomly assigned to one of the three groups. The generation of an unpredictable randomized allocation sequence will be completed using randomization.com through the use of a random allocation scheme (one block, size 120) with a 1:1:1 allocation ratio between the three groups. This randomization procedure was chosen to avoid selection bias with predictable random assignments using smaller block sizes in a non-blinded study design. This task will be completed by an individual that is not involved in the trial. In order to ensure allocation concealment, sequentially numbered, opaque, sealed envelopes (SNOSE) will be used. Once potential subjects have provided written, informed consent, they will sequentially draw an envelope and write their details on that envelope before opening to determine their group allocation. All envelopes will be similar in appearance and weight and cardboard will be placed within the envelope in order to make it impermeable to light.

### Treatment arms

**Figure 2:**
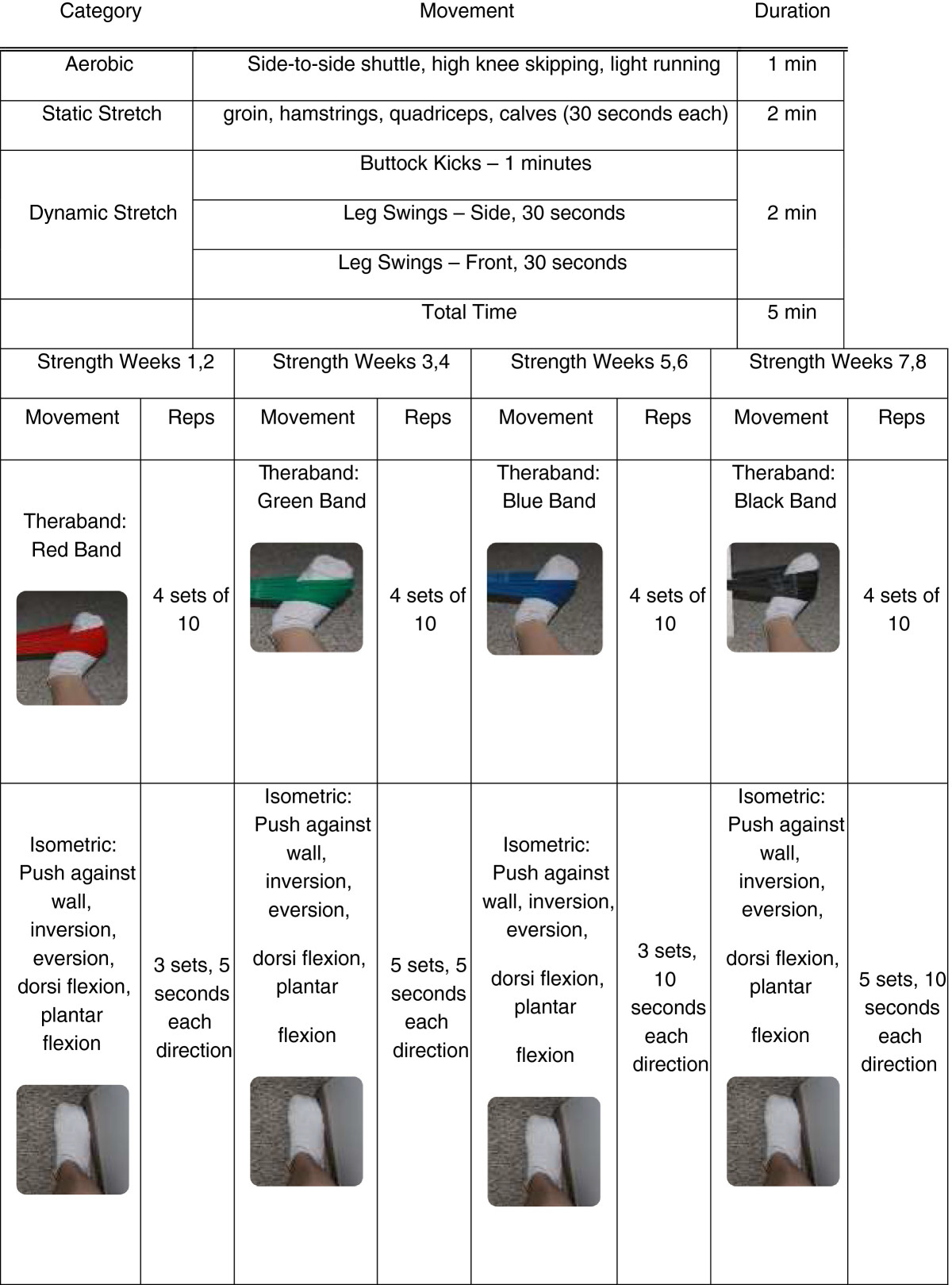
**Twenty-five minute training routine for the isolated ankle strengthening group.**

A home-based warm up routine consisting of aerobic activity, static stretching and dynamic stretching will be taught with recommended adherence by all three groups five times a week[41] The control group subjects will be asked to complete this warm up for twenty five minutes with no additional training (Table 1). The strength group and the balance group will be asked to complete specific training program components for 20 minutes in addition to a 5-minute warm up. The strength group will complete a training program using Thera-Band elastic bands[55] (Figure 2). The balance group will have training incorporating a Both Sides Up (BOSU) device with activities increasing in difficulty every two weeks[43, 46, 56] (Table 2). Subjects will meet in person with the study coordinator every two weeks to receive their new training exercises. All subjects will be asked to complete their respective routines five sessions per week. If the subject plans to run that day, they will be asked to complete their training immediately before their running session. If the subject does not plan to run that day, they are able to complete their respective training whatever time of day is convenient. Subjects will be asked to complete a physical activity log documenting the number of minutes completed for each exercise in their training routine as well as the number of minutes of running exposure on a weekly basis[42]. It is hypothesized that the common warm up will have no effect on muscular strength, lower extremity running mechanics, postural control, or injury prevention.

**Table 1:** **Twenty-five minute warm up routine for the activity matched control group**

Category	Movement	Duration
Aerobic	Side-to-side shuttle, high knee skipping, light running	5 min
Static stretch	groin, hamstrings, quadriceps, calves (30 seconds each)	10 min
Dynamic stretch	Buttock Kicks – 4 minutes	10 min
	Leg Swings – Side, 3 minutes	
	Leg Swings – Front, 3 minutes	
	Total Time	25 min

**Table 2:** **Twenty-five minute training routine for the functional balance training group**

Category		Movement				Duration	
Aerobic		Side-to-side shuttle, high knee skipping, light running				1 min	
Static stretch		groin, hamstrings, quadriceps, calves (30 seconds each)				2 min	
Dynamic stretch		Buttock Kicks – 1 minutes				2 min	
		Leg Swings – Side, 30 seconds					
		Leg Swings – Front, 30 seconds					
		Total time				5 min	
Balance weeks 1,2		Balance weeks 3,4		Balance weeks 5,6		Balance weeks 7,8	
Movement	Reps	Movement	Reps	Movement	Reps	Movement	Reps
Lunges: forward and backward	10 per side	Lunges: round the clock	5 per side	Lunge forward onto BOSU ball and backwards off of BOSU ball	10 per side per condition	Lunge forward onto BOSU ball and backwards off of BOSU ball	20 per side per direction
Squat: bipedal, box touch	10	Squat: bipedal on BOSU, ball facing down	10	Squat: single leg	10 per side	Squat: single leg mini squat on BOSU ball, ball facing up	10 per side
Hop in a box formation: bipedal	5 per direction	Hop in a box formation: single leg	3 per side per direction [6 per side in total]	Hop onto BOSU: Bipedal, ball facing upwards	10	Hop onto BOSU: single leg, ball facing upwards, forward, and side	5 per side per direction
Single leg stance: eyes open, eyes closed, variations with lower limb movements for increasing difficulty	5 per visual condition, 30 sec	Single leg stance: eyes open, eyes closed, variations with lower limb and trunk movements for increasing difficulty	5 per visual condition, 30 sec	Single leg stance on BOSU: ball facing upwards	5 per visual condition, 30 sec	Single leg stance on BOSU: ball facing upwards, variations in arm and torso movements.	5 per visual condition, 30 sec
Lateral Jump: left to right side	20 per side	Star Jump: left to right side	5 per side	Star jump: right leg to right leg, left leg to left leg	3 per side	Star jump: right leg to right leg, left leg to left leg, increasing jumping distance	3 per side

#### Measurements

Baseline characteristics will be collected for each subject including height, mass, running frequency, running experience, and injury history. Subjects will attend one testing session prior to the training intervention and one testing session immediately following the training intervention (within 9 weeks from baseline) for biomechanical data collection. These testing sessions will involve the collection of isokinetic strength data and the collection of lower extremity running mechanics (kinematics and kinetics) and postural control (dependent variables). Postural control will be assessed as balance deficits have previously been shown to be predictive of future ankle sprains in healthy athletes[57–59].

### Isokinetic strength

As running is dynamic, isokinetic measurements of strength will be completed. Isokinetic strength will be measured using a Biodex System 3 dynamometer (Biodex Medical System Inc, New York, NY, USA) to assess average peak torque for ankle eversion/inversion strength, ankle plantar flexion/dorsiflexion strength, knee flexion/extension strength and hip ab/adduction strength. For each movement, three maximal repetitions of concentric-concentric contractions will be completed. Three submaximal contractions will be allowed prior to three maximal contractions to allow for familiarization with the procedure. Isokinetic dynamometer results have previously been shown to produce reliable measurements for isokinetic strength[60, 61]. For ankle testing measurements, subjects will be placed in a semi recumbent position with 60° of knee flexion. Strength measurements will be made at 30°/s. For knee flexion/extension isokinetic strength, subjects will be placed in an upright seated position with knees and hips flexed to 90°[62]. Strength measurements will be made at 60°/s. For hip ab/adduction isokinetic strength, subjects will be standing in a vertical position and measurements will be made at 30°/s. Consistent verbal encouragement will be given in order to promote maximal effort.

### Postural control

To assess postural control, subjects will be asked to complete three 70-second single limb stance trials with their eyes open and three 45-second single limb stance trials with their eyes closed standing on a force platform embedded in the laboratory floor (Kistler Instruments AG, Winterthur, Switzerland). Only the dominant limb will be used as assessed using the following three tasks (2 out of 3): preferred kicking limb, step up test, and balance recovery[63]. The magnitude of movement and the temporal structure of the center of pressure (COP) trace will be analyzed. The magnitude of movement will be quantified using the path length and 95% ellipse area. The temporal structure will be quantified using the entropic half-life [E(1/2)] of the COP data in the medio-lateral and anterior-posterior direction[64, 65]. Additionally, postural control will be assessed using the Star Excursion Balance Test (SEBT), which has previously been shown to be a reliable and valid measure of postural control[66, 67].

### Kinematics and kinetics

To assess lower extremity running mechanics, subjects will perform 20 over-ground running trials (3.5 m/s ± 15%). Additionally, subjects will perform five minutes of treadmill running at their preferred speed following a five minute walking warm up at 2.5 mph. For the over ground running trials, kinematic data will be collected using an eight high-speed video camera system (Motion Analysis Corporation, Santa Rosa, CA, USA) at a sampling rate of 240 Hz. Previous studies have found that motion capture systems provide results with clinically acceptable errors and relatively high reliability depending on the accuracy of marker placement[68]. Three-dimensional marker traces will be reconstructed using Expert Vision Three-Dimensional Analysis software (Motion Analysis Corporation, Santa Rosa, CA, USA). Kinetic data will be collected simultaneously using a force plate (Kistler Instruments AG, Winterthur, Switzerland) embedded within the laboratory floor with a sampling frequency of 2400 Hz. Retro-reflective markers will be mounted on the rearfoot, shank, and thigh of the right lower extremity and the pelvis to measure three dimensional movements of the ankle, knee, and hip joints[69]. Markers will be placed over the right greater trochanter, medial and lateral knee joint axis, and medial and lateral malleoli in order to define joint centers. Position data from a static neutral trial will be collected in order to define the segment coordinate system. Joint markers will be removed for the running trials. For the treadmill walking, 26 retro-reflective markers were skin-mounted the arms, torso, pelvis, legs and feet. Markers were placed on both sides of the body (three on each foot, three on each shank, three on each thigh, one on each greater trochanter and lateral knee joint center, four on the pelvis) as well as two on the sternum, one on the spine (C7), and one on each shoulder. All biomechanical testing sessions will be completed by the same researcher in order to ensure maximum reliability.

### Injury assessment

Participants will be asked to complete their allocated treatment two days per week before their running sessions for maintenance following the initial 8 weeks of training. The primary outcome measure for this portion of the research project will be running-related injuries. During the 6-month follow up period, running injuries will be tracked and documented for each participant using StudyTRAX, a web-based Electronic Data Capture system. Injuries will be identified using the “any physical complaint” definition as “any physical complaint developed in relation to running activities and causing restriction in running distance, speed, duration, or frequency”[70, 71]. Injuries reports will be completed weekly using the questionnaire that has previously been validated for overuse injuries[72]. An additional question will be added to this questionnaire asking the participant if they experienced any “physical complaint that prevented them from being fully able to participate in running activities”[72, 73]. If a participant answers “yes” to the final question, they will be contacted directly and asked to fill out the “time-loss” injury questionnaire using a “time-loss injury” questionnaire that was adapted for running injuries[74]. All “time-loss” injuries occurring during this time will be assessed by a physiotherapist. Injury incidence and severity for overuse injuries will be quantified by the average weekly incidence and severity scores from the overuse injury questionnaire[72]. Subjects will be asked to complete a physical activity log documenting their running routines as the number of minutes of running exposure on a weekly basis[42]. This will provide information regarding the running frequency per week as well as the length of time of each run in minutes. Due to funding limitations, heart rate monitors and GPS tracking were not available. Therefore, no information will be available regarding the type of run (tempo, steady state, intervals). Injury incidence and severity for acute injuries will be quantified by injury rate (number of injuries/1000 running hours) and the number of days taken away from running due to the injury[72]. Participants will also self-report their protocol adherence every week during the eight week intervention[75].

#### Analysis

Descriptive statistics of baseline characteristics of the participants in each group will be reported (mean, standard deviation, 95% confidence intervals). These will be presented in tabular form in order to determine if the groups were well balanced with respect to age, mass, height, weekly mileage and running experience[76].

For the isokinetic strength measurements, the peak torque of the three maximal isokinetic contractions will be identified. For postural control, the average entropic half-life in the medio-lateral and anterior-posterior direction will be calculated for the eyes open and eyes closed single limb tasks in the ML and AP direction. Additionally, the average COP 95% ellipse area, average COP path length, and average SEBT reach distance will be calculated. For the over ground running trials, a discrete analysis will be completed using Kintrak software (Human Performance Laboratory, Calgary, Canada) to calculate three-dimensional joint angular displacements, and moments for the ankle, knee and hip joints. Specifically, peak ankle eversion/inversion angle and moment, peak knee ab/adduction angle and moment, peak knee internal/external rotation angle and moment, peak hip ab/adduction angle and moment and peak hip internal/external rotation angle and moment during the stance phase will be calculated. All variables will be normalized to the stance phase of the right foot where heel contact and toe off will be identified as crossing a 15 N threshold. Additionally, a vector-based analysis approach (iterative support vector machine [SVM]) will be used to determine the classification rate of subjects following training as well as to identify the movements that have changed following training using the entire data set rather than discrete time points for the treadmill run[77]. A multivariate linear regression will be used to determine whether the change from baseline to the end of training in each variable for each training group is significantly different from the change found in the control group while controlling for age, gender, and any other baseline differences between the groups.

Acute and overuse injury rates will be quantified separately due to the difference in injury definition[78]. Injury rates (number of injuries/1000 running hours) and 95% confidence intervals will be estimated. In an attempt to avoid the effects of dropout and adherence, an intent-to-treat analysis will be used. A multivariate Poisson regression analysis will be used to estimate incidence rate ratio (IRR) between the functional balance training group and the control group as well as between the isolated ankle strengthening group and the control group while controlling for age, gender and any other differences in baseline characteristics. Descriptive statistics for the amount of time missed from running due to injury (mean and standard deviation) will be calculated for both groups[79].

## Discussion

Reducing the likelihood of running-related injuries will have a widespread impact due to the popularity of recreational running. The effects of running injuries go beyond the short-term pain suffered by the individual[80]. Long term effects of injury include a reduction in the participation in sport, future joint health concerns such as osteoarthritis, and increased health care costs[15]. Avoiding injury will allow Canadians to remain active and enjoy the benefits of participating in aerobic activities in addition to reducing the healthcare costs associated with running injuries. The results from this study will be highly relevant for the field of health and wellness. This study will provide valuable information regarding the efficacy in using an 8-week isolated ankle strengthening program or an 8-week functional balance training program to reduce intrinsic risk factors for running injuries and the resulting injury rate in novice runners. Currently, the literature is lacking with respect to efficacy of training protocols for altering running injury rates.

The training protocols used in this study are very simple and could be used at home rather than requiring the assistance of a physical therapist. Additionally, they are short in duration and can easily be added to the warm-up period prior to a running routine. Thus, the training protocols should have relatively high adherence due to their ease of use. Successful results from this study would provide a major contribution for the potential in reducing running injuries.

## Authors’ information

Dr. Benno Nigg is a world renowned biomechanist with a long and successful career related to running and running injury. He is also the Co-Director of the Human Performance Laboratory at the University of Calgary and is the primary supervisor for Ms. Jennifer Baltich. Dr. Carolyn Emery is an associate professor, physiotherapist and sport injury epidemiologist. Dr. Emery is also the Associate Dean of Research in the Faculty of Kinesiology and the co-chair of the Sport Injury Prevention Research Centre (SIPRC) at the University of Calgary. Dr. Darren Stefanyshyn is a registered professional engineer and a leading biomechanist in the field of sport biomechanics. He is the current Associate Dean of Graduate Studies at the Faculty of Kinesiology of the University of Calgary.

## Authors’ original submitted files for images

Below are the links to the authors’ original submitted files for images. Authors’ original file for figure 1 Authors’ original file for figure 2
