# Supplementary figures and images for: The effects of isolated ankle strengthening and functional balance training on strength, running mechanics, postural control and injury prevention in novice runners: design of a randomized controlled trial
Source: BMC Musculoskelet Disord. 2014 Dec 4;15:407. doi: 10.1186/1471-2474-15-407 (PMC4295291; doi:10.1186/1471-2474-15-407)

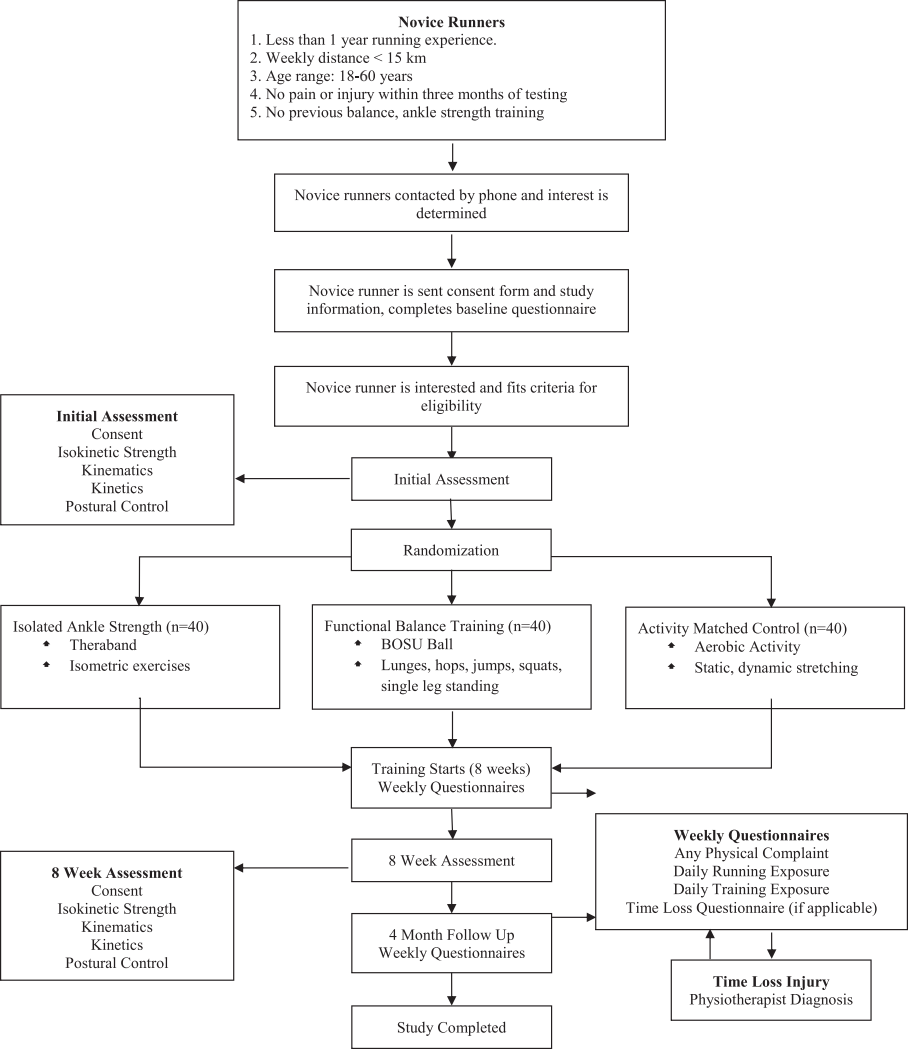

Supplement: Supplementary file 1 — Authors’ original file for figure 1 [file 12891_2014_2391_MOESM1_ESM.pdf]
