# Supplementary material for: The effects of isolated ankle strengthening and functional balance training on strength, running mechanics, postural control and injury prevention in novice runners: design of a randomized controlled trial
Source: BMC Musculoskelet Disord. 2014 Dec 4;15:407. doi: 10.1186/1471-2474-15-407 (PMC4295291; doi:10.1186/1471-2474-15-407)
Supplement: Supplementary file 2 — Authors’ original file for figure 2 [file 12891_2014_2391_MOESM2_ESM.pdf]

Category	Movement	Duration
Aerobic	Side-to-side shuttle, high knee skipping, light running	1 min
Static Stretch	groin, hamstrings, quadriceps, calves (30 seconds each)	2 min
Dynamic Stretch	Buttock Kicks – 1 minutes	2 min
	Leg Swings – Side, 30 seconds	
	Leg Swings – Front, 30 seconds	
	Total Time	5 min

Strength Weeks 1,2		Strength Weeks 3,4		Strength Weeks 5,6		Strength Weeks 7,8	
Movement	Reps	Movement	Reps	Movement	Reps	Movement	Reps
Theraband: Red Band 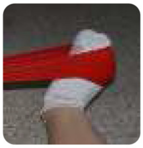	4 sets of 10	Theraband: Green Band 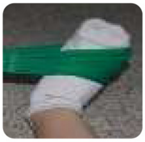	4 sets of 10	Theraband: Blue Band 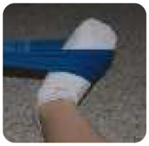	4 sets of 10	Theraband: Black Band 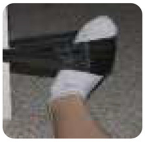	4 sets of 10
Isometric: Push against wall, inversion, eversion, dorsi flexion, plantar flexion 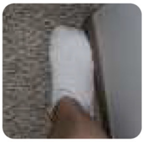	3 sets, 5 seconds each direction	Isometric: Push against wall, inversion, eversion, dorsi flexion, plantar flexion 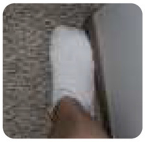	5 sets, 5 seconds each direction	Isometric: Push against wall, inversion, eversion, dorsi flexion, plantar flexion 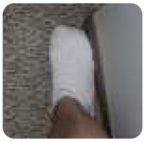	3 sets, 10 seconds each direction	Isometric: Push against wall, inversion, eversion, dorsi flexion, plantar flexion 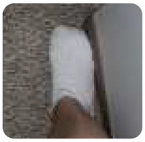	5 sets, 10 seconds each direction
